# The impact of cochlear implant microphone settings on the binaural hearing of experienced cochlear implant users with single-sided deafness

**DOI:** 10.1007/s00405-020-06450-5

**Published:** 2020-11-03

**Authors:** Anja Kurz, Maren Zanzinger, Rudolf Hagen, Kristen Rak

**Affiliations:** Department of Oto-Rhino-Laryngology, Plastic, Aesthetic and Reconstructive Head and Neck Surgery, Comprehensive Hearing Center, University Hospital Würzburg, Josef Schneider Str. 11, 97080 Würzburg, Germany

**Keywords:** Single-sided deafness, Cochlear implant, Adaptive directional microphone setting, Questionnaire

## Abstract

**Objective:**

Cochlear implantation has become a well-accepted treatment option for people with single-sided deafness (SSD) and has become a clinical standard in many countries. A cochlear implant (CI) is the only device which restores binaural hearing. The effect of microphone directionality (MD) settings has been investigated in other CI indication groups, but its impact on speech perception in noise has not been established in CI users with SSD. The focus of this investigation was, therefore, to assess binaural hearing effects using different MD settings in CI users with SSD.

**Methods:**

Twenty-nine experienced CI users with SSD were recruited to determine speech reception thresholds with varying target and noise sources to define binaural effects (head shadow, squelch, summation, and spatial release from masking), sound localization, and sound quality using the SSQ12 and HISQUI_19_ questionnaires. Outcome measures included the MD settings “natural”, “adaptive”, and “omnidirectional”.

**Results:**

The 29 participants involved in the study were divided into two groups: 11 SONNET users and 18 OPUS 2/RONDO users. In both groups, a significant head shadow effect of 7.4–9.2 dB was achieved with the CI. The MD setting “adaptive” provided a significant head shadow effect of 9.2 dB, a squelch effect of 0.9 dB, and spatial release from masking of 7.6 dB in the SONNET group. No significant summation effect could be determined in either group with CI. Outcomes with the omnidirectional setting were not significantly different between groups. For both groups, localization improved significantly when the CI was activated and was best when the omnidirectional setting was used. The groups’ sound quality scores did not significantly differ.

**Conclusions:**

Adaptive directional microphone settings improve speech perception and binaural hearing abilities in CI users with SSD. Binaural effect measures are valuable to quantify the benefit of CI use, especially in this indication group.

## Introduction

Cochlear implant (CI) provision is now an accepted treatment in Europe for many people with single-sided deafness (SSD). The reported benefits of CI use include significantly better speech understanding in noise, improved localization abilities, reduction of tinnitus, improved self-esteem, and less fatigue [1–7]. The challenge in this patient group is the integration of electric input (from the CI) with acoustic hearing (from the contralateral ear) to enable the restoration of binaural hearing [8–10].

A consensus treatment road map for SSD patients was published in 2017. It suggested offering a contralateral routing of signal (CROS) and bone conduction (BCD) hearing aid trial prior to implantation [11, 12]. This consensus paper also recommended reporting on the same outcome measures across centers to ensure comparability [12]. If the CROS and BCD devices do not provide acceptable hearing, CI provision is recommended. However, since providing CI recipients with the best possible hearing is our goal, determining which audio processor can provide users with the best objective and subjective hearing is of obvious clinical interest.

Since 2009, our institution has been providing people with SSD with a CI. Before 2014, the standard audio processor at our institution was the OPUS2 (MED-EL) which, as per the technology at that time, has an omnidirectional microphone. When the SONNET audio processor (MED-EL) was introduced in 2014, it became our audio processor of choice, partly because it has microphone directionality settings designed to improve users’ speech understanding in noise and sound localization abilities. And indeed, the SONNET has been shown to improve speech understanding in challenging situations, improve sound quality, and increase user satisfaction [13–16]. These studies, however, were conducted on CI users (unilateral or bilateral) with bilateral severe-to-profound hearing loss or on CI/EAS (electric acoustic stimulation) users—not, to the best of our knowledge, on a group of CI users with SSD.

Since our institution has implanted a relatively large group of CI users with SSD who use either an OPUS 2 or a SONNET audio processor, we are in the position fill this research gap by assessing the level of speech understanding and sound localization that each audio processor can provide. That is our primary aim. Our secondary aim was to determine the factors that influence who would benefit from an upgrade to the SONNET earlier during our clinical routine. Finally, for SONNET users with SSD, we assessed how using different microphone directional settings affected their hearing performance.

## Materials and methods

This prospective experimental study was performed with the approval of the Ethics Committee of the Medical University of Würzburg, Germany. All procedures performed in this study involving human participants were in accordance with ethical standards of the hospital and with the Declaration of Helsinki (2013).

### Inclusion criteria

SSD is defined as a pure-tone average (PTA) of ≥ 70 dB HL in the poorer ear and of ≤ 30 dB HL in the better ear (interaural threshold gap ≥ 40 dB HL), with PTA referring to the mean threshold at pure-tone frequencies of 0.5, 1, 2, and 4 kHz [12]. People with SSD who received a CI at our institution between 2009 and 2016 (inclusive) were eligible to participate in the study. These patients were informed about the study and invited to participate; most declined due to time constraints. Among these patients, some had not come to their regularly scheduled annual appointments for years and thus took this as an opportunity to have a check-up again. The last reported monosyllabic speech reception threshold in quiet (at 65 dB SPL) in the medical records was not a criterion for exclusion (see Table 1). The willingness to participate in the study served as inclusion criterion.

**Table 1:** Demographic data of all subjects

ID	Age (years)	Sex	DoD (years)	Ear with CI	Etiology	IE (years)	Sound processor	Implant	Electrode Array	Monosyllabic WRS @ 65 dB SPL with CI (%)	Unaided contralateral PTA
X1	59	F	0.5	L	CME	6	OPUS 2	SONATA	Standard	50	21.3
X2	60	F	Childhood	L	Unknown	7	OPUS 2	SONATA	Standard	25	15.0
X3	50	F	Childhood	R	Congenital HL	4	OPUS 2	CONCERTO	FLEXsoft	45	16.3
X4	54	F	40	R	Sudden HL	5	RONDO	SONATA	FLEXsoft	60	8.8
X5	70	M	11	L	Head trauma	5	RONDO	SONATA	FLEXsoft	50	20.0
X6	57	M	9	L	Unknown	2	SONNET	SYNCHRONY	FLEXsoft	55	12.5
X7	49	F	8	L	Unknown	5	SONNET	SONATA	FLEXsoft	75	22.5
X8	60	F	8	R	Otosclerosis/surgeries	8	SONNET	SONATA	Standard	50	8.8
X90	34	M	0.1	L	Head trauma	7	OPUS 2	SONATA	Standard	60	8.8
X10	60	F	1.5	R	Ototoxic medication	3	SONNET	SONATA	FLEXsoft	0	18.8
X11	61	F	44	L	OME	4	OPUS 2	SONATA	FLEXsoft	15	16.3
X12	52	F	Childhood	R	Mumps	4	OPUS 2	SONATA	FLEXsoft	45	15.0
X13	52	F	6.5	L	Post-surgery	2	OPUS 2	SONATA	Standard	25	11.3
X14	59	F	1	R	Sudden HL	3	SONNET	SYNCHRONY	Flex28	65	10.0
X15	33	M	1	R	Several surgeries	4	OPUS 2	SONATA	FLEXsoft	95	18.8
X16	57	F	1.5	R	Sudden HL	4	SONNET	SONATA	FLEXsoft	10	12.5
X17	38	M	0.2	R	Head trauma	8	SONNET	SONATA	Standard	40	26.3
X18	55	M	1	R	Sudden HL	6	OPUS 2	SONATA	FLEXsoft	40	5.0
X19	56	F	0.75	R	Sudden HL	5	OPUS 2	SONATA	FLEXsoft	25	10.0
X20	66	M	6	R	Sudden HL	4	SONNET	SONATA	FLEXsoft	35	8.8
X21	25	F	20	L	OME	4	SONNET	SONATA	FLEX24	20	8.8
X22	27	M	1	R	Sudden HL	5	SONNET	SONATA	FLEX24	50	5.0
X23	25	W	1	L	Sudden HL	5	RONDO	CONCERTO	FLEXsoft	25	7.5
X24	21	W	6	L	Unknown	5	OPUS 2	CONCERTO	FLEXsoft	60	5.0
X25	56	W	47	L	Unknown	6	SONNET	SONATA	FLEXsoft	55	3.8
X26	26	M	21	R	Unknown	5	OPUS 2	CONCERTO	FLEXsoft	65	13.8
X27	57	W	4	R	Progressive HL	4	OPUS 2	SONATA	FLEXsoft	35	13.8
X28	60	W	7	R	Surgeries	5	RONDO	SONATA	Standard	30	8.8
X29	62	M	10	R	Sudden HL	6	OPUS 2	SONATA	FLEXsoft	60	5.0

*DoD* duration of deafness, *IE* implant experience, *L* left, *R* right, *CME* chronic mesotympanic otitis, *HL* hearing loss, *OME* otitis media with effusion, *WRS* word recognition score, and *PTA* pure-tone average

### CI audio processor settings

The OPUS 2 and the RONDO have one omnidirectional microphone. The SONNET, in contrast, has the two omnidirectional microphones, which enables microphone directionality. The three microphone modes that can be programmed by the audiologist in the SONNET are (1) “natural mode”, in which the two microphones are combined to form directionality towards the front in the higher frequencies, and omnidirectionality in the lower frequencies; (2) “adaptive mode”, in which both microphones are combined with a focus towards the front, adaptively suppressing noise from the side and from behind; and (3) “omnidirectional mode”, which provides the same directionality (or lack thereof) as previous audio processor generations (equipped with only one microphone) [17]. An overview of the corresponding polar plots can be found in the literature [14, 17].

All participants were tested using the SONNET’s default settings: coding strategy FS4; Wind Noise Reduction (mild setting); Compression Ratio, 3:1; Adaptive Directionality mode: always directional. The following microphone directionality settings for SONNET were used: program 1, natural mode; program 2, adaptive mode; program 3, omnidirectional mode.

The OPUS 2 was programmed using the FS4 coding strategy with one exception (one participant used HDCIS), compression Ratio 3:1, and was not reprogrammed for the purpose of the actual investigation.

Each participant used his or her own audio processors during testing. Prior to testing, all processors were carefully checked to ensure proper functionality.

### Study design

First, participants completed two questionnaires (SSQ12 and HISQUI_19_). Second, speech perception and sound localization testing were conducted.

### Questionnaires

Participants subjectively assessed their daily hearing function via the 12-item version of the Speech, Spatial, and Qualities of Hearing Scale (SSQ12) questionnaire [18]. As its title indicates, the SSQ12 had 12 questions, which are divided into three subjections: speech, spatial, and sound qualities. Each question is answerable on a Likert scale (0–10) in which 0 means “not at all” (= absolute disability) and 10 means “perfectly” (= complete ability); the higher the scores, the greater the ability.

The hearing implant sound quality index (HISQUI_19_) was used to determine self-perceived sound quality [19]. The HISQUI_19_ consists of 19 questions aimed at determining overall sound quality ranging from < 30 (very poor) to the maximum score of 133 (excellent). Less than 30 points indicates very poor sound quality, 30–60 points indicates a poor sound quality, 60–90 points indicates a moderate sound quality, 90–110 points indicates good sound quality, and above 110 points indicates a very good sound quality.

### Speech perception and sound localization

Speech perception and sound localization testing were conducted in a sound-isolated chamber (3.15 × 3.10 × 2.10 m with a reverberation time T_60_s at 500 Hz) using active loudspeakers (M52 Klein & Hummel, Georg Neumann GmbH, Berlin, Germany) and a pre-amplification system (DigiMax D8, FireStudio, PreSonus Audio Electronics Inc., Baton Rouge, LA, USA). A custom program using the software Matlab (Math Works, Natrick, MA, USA) was used to conduct speech perception and localization tests. The participants were seated in the center of a semi-circle of nine loudspeakers, equally spaced in the frontal horizontal plane, with a radius of 1.5 m.

Speech perception in quiet scores were taken from participants records. These were reviewed to ensure that the participant groups had a similar hearing ability, not to evaluate differences in audio processor performance. Perception in quiet was assessed via word recognition scores (Monosyllables) in quiet at 65 dB SPL. Participants were tested with their CI on and their contralateral (normal-hearing) ear masked using earplugs and earmuffs or insert earphones with constant noise, as per clinical norm. This testing was not part of the study, but was included to show that participants groups matched. The OPUS2/RONDO and SONNET group were matched in terms of age, duration of unilateral hearing loss, hearing experience with CI, and monosyllabic word recognition score in quiet.

**Fig. 1:**
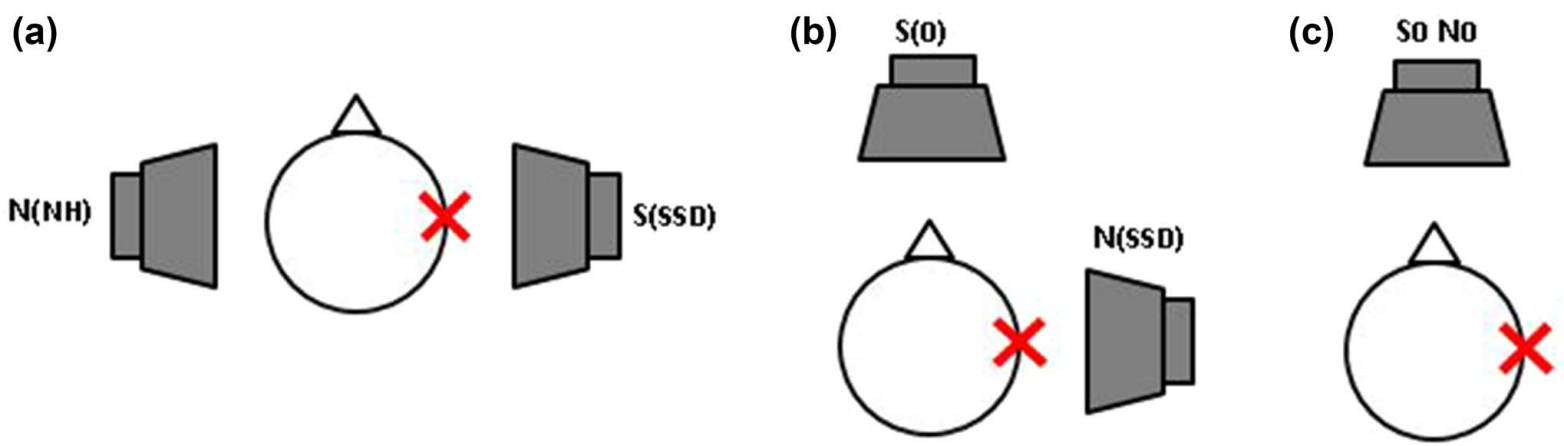
Spatial configurations for speech-in-noise testing in the case of single-sided deafness (here in the right ear, represented by the X). N represents the “noise” and S represents the “speech” signal. The configurations are used to determine the **a** head shadow effect, **b** squelch effect, and **c** summation effect

Speech perception in noise was assessed via the German language Oldenburg sentence test (OLSA). Testing was conducted in different spatial configurations with a constant masker (OLSA noise) at 65 dB SPL and a variable speech signal (see Fig. 1). The measured SRT quantifies speech perception in noise, resulting in dB signal-to-noise (SNR) ratio that is required for 50% of words to be correctly understood [20]. To ensure the participants understood how the test works, each participant completed two training lists (20 sentences in randomly selected directionality settings) before testing began. These results were discarded.

In the evaluated measurements, participants completed one list with 30 sentences. The order of the presentations and the test lists were randomized to minimize the effects of training and fatigue. Three spatial configurations were tested: (a) the signal was presented to the deaf side, while noise was presented to the normal-hearing side (S_SSD_N_NH_); (b) the signal was presented from the front (S0), while noise was presented to the deaf side (S_0_N_SSD_); (c) the signal and noise were both presented from the front (S_0_N_0_) (see Fig. 1a–c). The SRT was measured in both aided (with the CI on) and unaided (with the CI off) conditions in the described spatial configurations, which resulted in the calculation of the following binaural effects:

- the head shadow effect, which is the attenuation that occurs when a signal addressed to one ear must travel around the head to reach the other ear
- binaural squelch, is hearing advantage derived from hearing with both ears, even if one of the ears has a worse SNR
- binaural summation, which is a beneficial redundancy effect that occurs when the signal is presented to both ears instead of only one ear
- spatial release from masking (SRM), which is the benefit derived from spatially separating the target sound from competing (e.g., background) noise.

A detailed description of the calculation procedure can be found in the SSD consensus protocol [12].

To test the localization abilities, the localization stimuli and procedure as recommended by Van de Heyning et al. were implemented [12]. In our set-up, nine loudspeakers equally distributed between − 90° (left) and + 90° (right) azimuth and an angle between two nearby loudspeakers of 22.5° were used. Two noise stimuli with two spectral shapes [Comité Consultatif International Téléphonique et Télégraphique (CCITT) noise stimuli using the ipsi- and contralateral head-related transfer function)] and a duration of 1 s (rise and fall times of 20 ms each) were presented randomly at 65, 70, and 75 dBA [21]. This resulted in 54 presentations (nine loudspeakers × 3 levels × 2 signals) in the test condition. No training prior to testing was conducted and the participants were instructed not to turn their heads, while the stimulus was presented. Like speech-in-noise testing, the order of testing was pseudo-randomized. The localization performance was calculated using the total root-mean-square error (RMSE); therefore, lower scores indicated better localization ability [22].

### Statistical analysis

Statistical analysis and generation of figures were performed using GraphPad Prism 8.0 and InStat 3 (both GraphPad Software, San Diego, CA, US). Due to the small sample size, quantitative data are reported as median and range (minimum and maximum). The data distribution was checked using the Kolmogorov–Smirnov test. Paired Student *t* tests, two tailed, were used whenever normal distribution was confirmed. The Mann–Whitney test was applied for group comparisons (speech in noise, questionnaires). For analysis of the localization results, repeated-measures analysis of variance using the mixed-effects analysis with the Tukey´s correction factor for post hoc analysis was used. Significance was set to < 0.05.

## Results

### Participants

Sixty-nine potential participants met the inclusion criteria and were invited to participate in this study. Of these 69 people, 29 agreed to participate (14 OPUS 2 users, 11 SONNET users, and 4 RONDO users). The RONDO users were included (in the OPUS 2 group), because the RONDO audio processor uses the OPUS 2 technology and, like the OPUS 2, has 1 microphone (therefore is also omnidirectional). Furthermore, for CI users with SSD, speech perception scores with OPUS 2 and the RONDO are not significantly different when sound/noise is presented laterally or from the front (4).

The OPUS 2/RONDO group had a median age of 49.2 years (range 21‒70 years) and a median duration of unilateral hearing loss of 17.5 years (range 0.1‒60 years). The SONNET group had a median age of 50.4 years (range 25‒66 years) and a median duration of unilateral hearing loss of 10.3 years (range 0.2‒47 years). Both groups had a median CI experience of 5 years (range 2‒8 years). The median monosyllabic word recognition score in quiet was 45% (range 15%‒95%) for the OPUS 2 group and 50% (range 0‒75%) for the SONNET group. No significant difference was found between groups for age, duration of unilateral hearing loss, hearing experience with a CI, or monosyllabic word recognition in quiet (Friedman test, *p* > 0.05). See Table 1 for participants’ demographic details.

### Speech in noise performance

See Table 2 for the speech understanding scores for the SONNET and OPUS 2/RONDO groups with each microphone setting and at each speech/noise set-up.

**Table 2:** Median speech reception thresholds and ranges (in dB SRT) for each audio processor and setting for the measured loudspeaker configurations

	SONNET				OPUS 2/RONDO	
	Unaided	Natural	Adaptive	Omnidirectional	Unaided	Omnidirectional
S_SSD_N_NH_	1.6 (0.5–8.4)	− 4.8 (− 9.8–3.6)	− 6.4 (− 14.4–1.0)	− 6.9 (− 11.1–9)	1.7 (− 3–6.3)	− 6.1 (− 11.1–4.4)
S_0_N_SSD_	− 11.8 (− 16.7 to − 9.9)	− 1.9 (− 13.9 to − 8.6)	− 13.1 (− 20.8 to − 8.8)	− 12.4 (− 16.4 to − 8.4)	− 11.6 (− 18.3 to − 8.6)	− 10.6 (− 16.2 to − 6.5)
S_0_N_0_	− 5.5 (− 6.6 to − 2.6)	− 5.6 (− 6.5 to − 3.7)	− 5.7 (− 6.6 to − 3.5)	− 5.6 (− 6.8 to − 3.9)	− 5.4 (− 9–2.2)	− 5.3 (− 9.3–2.2)

*S*_*SSD*_*N*_*NH*_* set-up* For the SONNET group, speech understanding was significantly better in each aided condition than in the unaided condition (all *p* < 0.01). No significant differences were found between the microphone settings in the aided condition [Mixed-Effects analysis with Tukey´s correction factor (Chi-square: 0.1041; df: 1; *p* < 0.01)]. For the OPUS 2/RONDO group, a significant difference was detected between the unaided and aided conditions (paired *T* test; *p* < 0.001).

*S*_*0*_*N*_*SSD*_* set-up* For the SONNET group, speech perception was significantly better with the adaptive setting than with the natural (*p* = 0.047) or the omnidirectional setting (*p* = 0.035). For the OPUS 2/RONDO group, speech perception was significantly better in the unaided setting than in the aided setting (*p* = 0.007), Paired T tests were used for comparisons.

*S*_*0*_*N*_*0*_* set-up* No significant differences between any of the tested conditions were found in either group [Mixed-effects model with Tukey’s correction factor (Chi-square: 17.57; df: 1; *p* < 0.0001)].

### Binaural effects

**Fig. 2:**
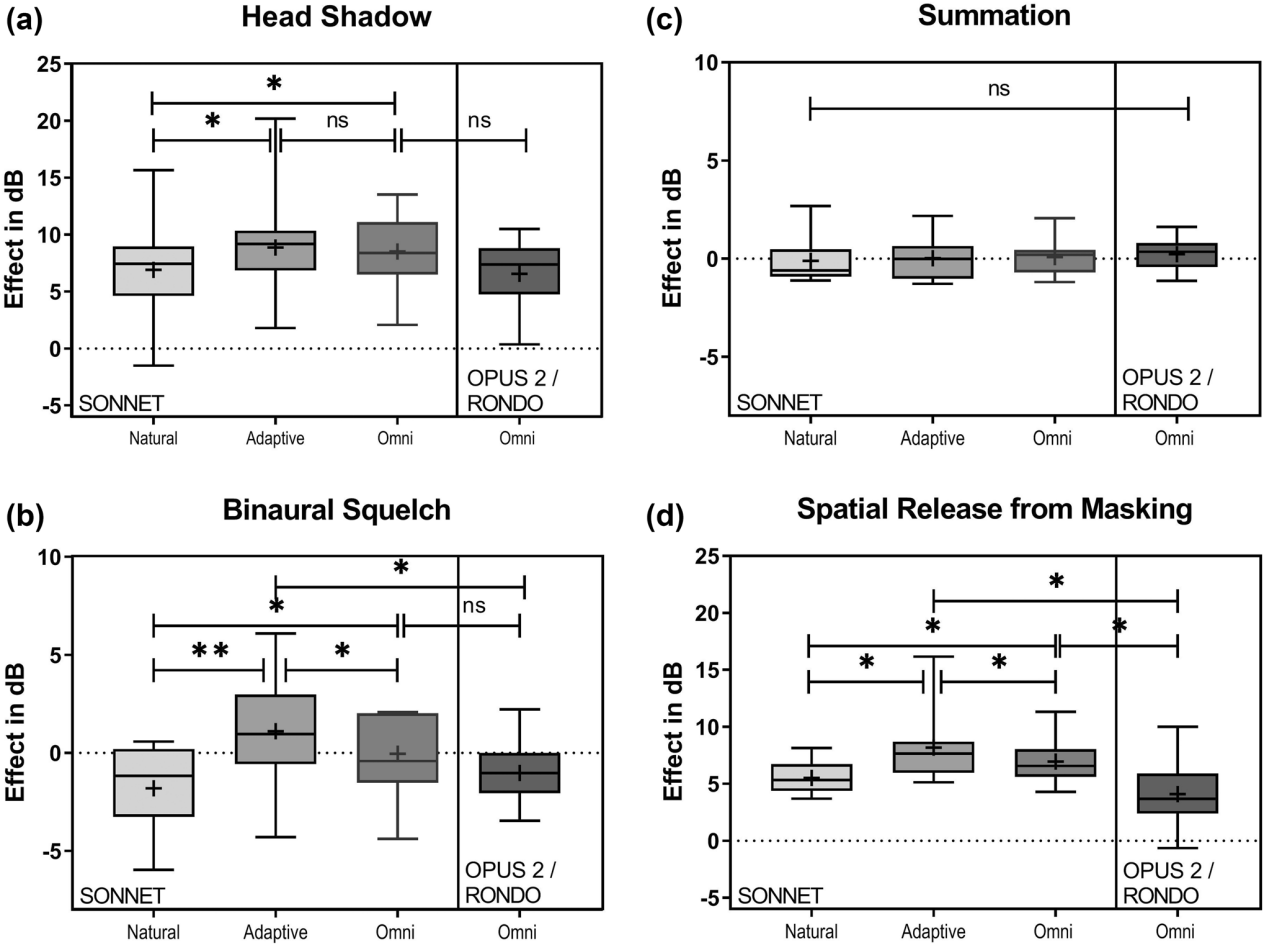
Binaural effects (**a**–**d**) in the different microphone directionality settings for the SONNET and OPUS 2/RONDO groups. Box-and-whisker plots denote minimum, quartile 1, median, quartile 3, and maximum values; + represents the mean; significant differences are denoted with *(*p* < 0.05) and **(*p* < 0.01), *ns* not significant. Higher scores indicate better performance

*Head shadow effect* With the SONNET, the head shadow effect was 7.4 dB (range − 1.5‒5.7) in natural mode, 9.2 dB SRT (range 1.8‒20.2) in adaptive mode, and 8.4 dB (range 0.1‒3.5) in omnidirectional mode. Compared to with the natural mode, scores with the adaptive mode (*p* = 0.0466) and with the omnidirectional mode (*p* = 0.0273) were significantly better. With the OPUS 2/RONDO, the head shadow effect was 7.4 dB (range 0.4‒10.5). No significant difference was found between this score and with the score with the SONNET in omnidirectional mode (see Fig. 2a).

*Binaural Squelch* With the SONNET, the binaural squelch effect was -1.2 dB (range − 5.9‒0.6) in natural mode, 0.9 dB (range − 4.3‒6.1), and in adaptive mode, and − 0.4 dB (range − 4.4‒2.5) in the omnidirectional mode. Scores with the adaptive mode was significantly better than the natural mode (*p* = 0.0470) or the omnidirectional mode (*p* = 0.0348). Scores were also significantly better with the omnidirectional mode than with the natural mode (*p* = 0.0116). With the OPUS 2/RONDO, the binaural squelch effect was − 1.0 dB (range − 3.5‒2.2). This was not significantly different than with the SONNET in omnidirectional mode, but it was significantly worse than with the SONNET adaptive mode (*p* = 0.0261) (see Fig. 2b).

*Binaural Summation* With the SONNET, the binaural summation effect was − 0.6 dB (range − 1.1‒2.7) in natural mode, 0.0 dB (range − 1.3‒2.2), and in adaptive mode, and 0.2 dB (range − 1.2‒2.1) in the omnidirectional mode. No significant differences were found between SONNET modes. With the OPUS 2/RONDO, the binaural summation effect was − 0.4 dB (range − 1.1‒1.6). No significant differences were found between SONNET modes and the OPUS 2/RONDO (see Fig. 2c).

*Spatial release from masking (SRM)* With the SONNET, SRM was 5.3 dB (range 3.7‒8.1) in natural mode, 7.6 dB (range 5.1‒16.1) in adaptive mode, and 6.5 dB (range 4.3‒11.3) in the omnidirectional mode. Scores were significantly better with the adaptive mode than the natural mode (*p* = 0.0470) and the omnidirectional mode (*p* = 0.0302). SRM was also significantly better with the omnidirectional than the natural mode (*p* = 0.0153). With the OPUS 2/RONDO, the SRM was 5.1 dB (range 2.3‒10.0). Compared to with the OPUS 2/RONDO, scores were significantly better with the SONNET in the adaptive mode (*p* = 0.001) and with the SONNET in omnidirectional mode (*p* = 0.005) (see Fig. 2d). Mann–Whitney tests were used for comparisons.

### Localization

For the SONNET group, localization performance was significantly better in all aided conditions compared to the unaided condition (*p* < 0.01). Localization was significantly better in the omnidirectional mode when compared to the adaptive mode [mixed-effects model with Tukey’s correction factor (Chi-square 1.281; df 1; *p* = 0.001)]. The median scores for each mode were as follows:

- Unaided: 74.3° (range 50.9‒106.2°);
- in the natural mode: 31.9° (range 19.0‒58.3°);
- in the adaptive mode: 27.3° (range 19.4‒77.9°);
- in the omnidirectional mode: 22.1° (range 16.6‒64.8°).

**Fig. 3:**
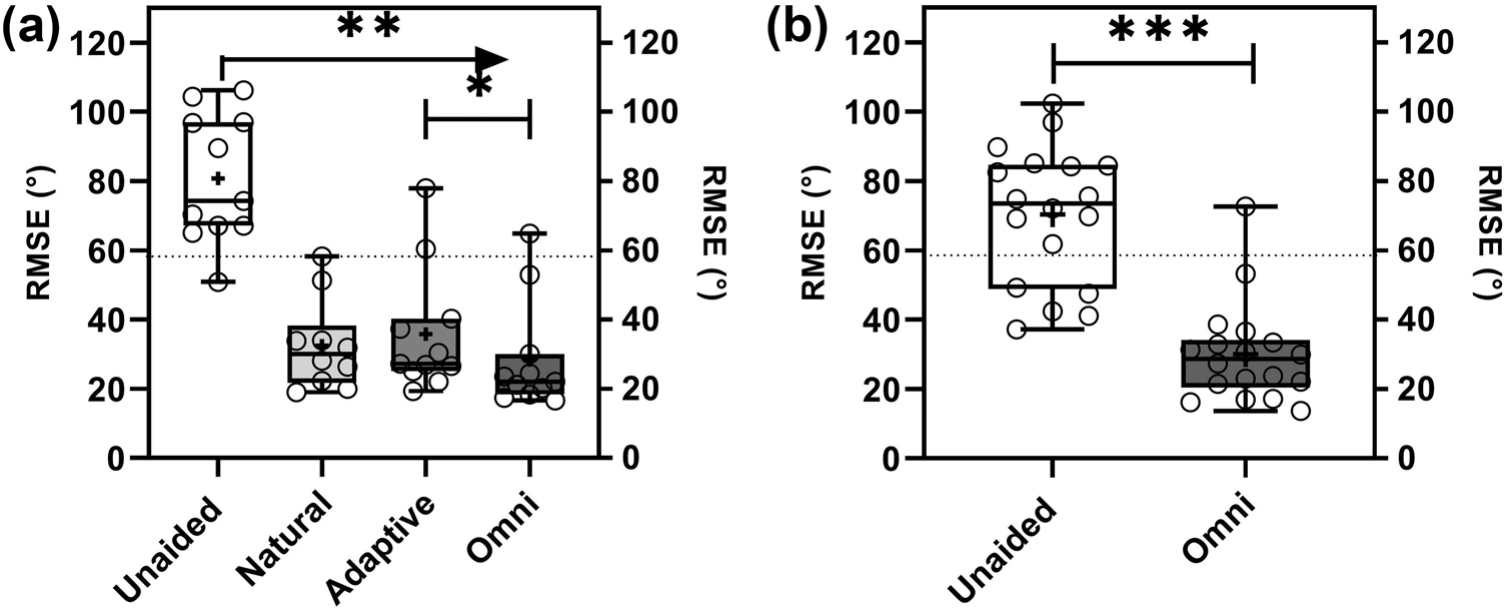
Box-and-whisker plots of RMSE in sound localization for the **a** SONNET and **b** OPUS 2/RONDO groups including minimum, quartile 1, median, quartile 3, and maximum; significant differences are * denoted with *(*p* < 0.05), **(*p* < 0.01), and ***(*p* < 0.001). Lower scores indicate better performance

For the OPUS 2/RONDO group, users had significantly better localization with their CI on (aided) than with their CI off (unaided) (paired *T* test; *p* < 0.001). The scores were as follows: 28.7° (range 13.7‒72.6°) aided and 73.5° (range 37.2‒102.3°) unaided (Fig. 3a, b).

### Subjective evaluation

See Table 3 for SSQ12 and HISQUI_19_ scores for each group.

**Table 3:** Median SSQ12 and HISQUI_19_ scores (and ranges) for each group. For both questionnaires, higher scores indicate better performance

	SSQ12			HISQUI_19_
	Speech	Spatial	Quality of hearing	
SONNET	7.4 (2.6‒9.1)	6 (1–10)	7.5 (2.7–10)	97 (34‒128)
OPUS 2/RONDO	6.7 (1.2–8.5)	5.8 (1.0–9.3)	7.2 (1.6–10)	94 (39‒121)

No significant differences were found between groups in either SSQ12 scores or HISQUI_19_ score using the Mann–Whitney test. HISQUI_19_ scores indicated that both groups had a “good sound quality” with CI use.

## Discussion

The results of this study indicate two key points: (1) using a CI with an adaptive microphone directionality setting improves CI users with SSD’s speech understanding in noise, and (2) when testing CI users with SSD, it is important to assess binaural effect measures to quantify the benefit, rather than only looking at SRT in noise. As speech-in-noise scores in the S_SSD_N_NH_ setting demonstrates, activating the CI will result in better hearing, regardless of the audio processor being used. This effect has been shown by other groups, e.g., [1, 10], and [22–28]. However, the scores on the S_0_N_SSD_ setting and on the S_0_N_0_ setting would appear to contradict this. In S_0_N_SSD_ setting, using microphone directionality did not significantly improve performance compared to turning the SONNET off. While, with the OPUS 2/RONDO, scores were significantly better with the CI off, though their clinical significance is open to question, since the difference was only 1 dB (which is a lesser difference than adaptive vs. unaided with the SONNET). In the S_0_N_0_ setting, there were no significant differences; in fact, all six scores were between − 5.3 and − 5.7.

The focus of this investigation was to determine if an adaptive microphone directionality setting would provide an additional benefit, this could only be confirmed by calculating binaural effects. To our best of knowledge, this is the first investigation in CI users with SSD that has evaluated different microphone directionality settings. As such, comparing the present results with the SONNET with those from the literature is problematic, because those results were obtained using an omnidirectional microphone [1, 10, 12, 22].

The results in the SONNET group show that the head shadow effect significantly increased when the adaptive microphone directionality setting was used. The head shadow effect in the SONNET group was also higher when using the omnidirectional setting than the natural setting. These findings are supported by results from De Ceulaer et al. [17], who reported a median SRT in noise in a group of adults with bilateral deafness and a unilateral CI was − 5.6 dB SNR with omnidirectional, − 9.1 dB SNR with natural, and − 12.8 dB SNR with adaptive microphone. Interestingly, the benefit of using the adaptive microphone setting is so strong; it is also evident in CI users with SSD. CI users with SSD are a special group, because electric hearing (with the CI) needs to be incorporated to acoustic hearing (with their contralateral ear). This process takes time and should be supported by postoperative rehabilitation and training using direct acoustic input [27]. Furthermore, binaural processing strategies evolve as the user gains experience with a CI, and thus, a longer period using the CI may be necessary before binaural squelch and summation effects can be fully realized [28].

Mertens et al. [3] showed that it took 12 months of CI use before the head shadow effect was significant. In addition, while no significant squelch effect could be detected over the long term, the SRM was significant after only 6 months after first fitting. It needs to be kept in mind when comparing data with those of Mertens’s et al. that most of their participants had incapacitating tinnitus, which is an intrusive factor that may have influenced their unaided results. Tinnitus did not influence results in the present study, although it is possible that the participants’ broad range of experience with a CI or the fact that they used their own audio processors during testing (possible habituation effect) did influence results. This could explain why our data showed a significant squelch effect and SRM. Contrary to Mertens et al., we could not detect a significant summation effect. This is also in line what other groups have found [10, 29].

In our investigation, we compared two different user groups that had comparable aided word recognition scores in quiet at 65 dB SPL. The only microphone setting that allows a comparison regardless of the audio processor is the omnidirectional setting. Comparing results of the omnidirectional setting in both groups revealed no significant differences except for the SRM, which was significantly higher (i.e., better) with the SONNET. As mentioned earlier, this effect could be due to a demographic difference between the groups. Other authors, e.g., Litovsky et al. [30] have suggested that this may be because the electric signal via the CI may not optimally integrate with the acoustic signal via the normal-hearing ear in every CI user with SSD. The reason for this less than optimal integration could be a frequency or temporal mismatch between the ears [31–33]. Looking at our results, we have a median duration of unilateral deafness of 17.5 years in the OPUS 2/RONDO group and 10.3 years in the SONNET 2 group. Both groups have a median CI experience of 5 years (range 2–8 years). Although the two groups in the present study have a median CI experience of 5 years, the OPUS 2/RONDO group has 7.2 more years of median deafness. Thus, it is possible that some of the participants have “achieved” optimal integration of electric to acoustic hearing, but others have not. Another explanation of the difference in SRM between the audio processors is that the SONNET has superior signal pre-processing, aligning frequency, and temporal processing [13].

More importantly, using the adaptive setting significantly improved the binaural squelch and SRM. When would anticipate that OPUS 2/RONDO users would do better if they had an adaptive microphone; an “upgrade” study wherein all OPUS 2/RONDO users are upgraded to the SONNET and thereby act as their own control, would need to be conducted to demonstrate this. This could be the topic of a future study.

Interestingly, we also find an impact on localization results when switching between microphone settings. Again, the MD settings seem to have a strong impact on the perception of our participants, demonstrating the effectiveness of microphone filtering. When using the omnidirectional microphone, our results show that the sound signal has the potential to be equally perceived in the tested semi-circle, while using the adaptive setting restricts the area of perception (see measured polar plots [17]). Comparing the RSME in the omnidirectional setting with both groups did not show a significant difference. Other groups using a comparable localization setting (− 90° to + 90°; using narrow-band noise, wind noise, or speech-shaped noise) and using an omnidirectional microphone setting reported an average RMS error between 27 and 32° [24, 29, 34–36]. This RMS error in the literature is poorer than our results with the omnidirectional microphone activated with the SONNET. The average RMS error in the OPUS 2 group was 28°, which clearly indicates that the above-mentioned studies were performed with an omnidirectional microphone and sound processors of an older generation. Only with the SONNET and different microphone settings did the RMS error decrease (in the natural mode: 31.9° (range 19.0‒58.3°); adaptive mode: 27.3° (range 19.4‒77.9°); in the omnidirectional mode: 22.1° (range 16.6‒64.8°). This can be explained by its in-built technology [13, 17] or the idea that binaural integration has taken place in our population. This might be due to the median hearing experience of 5 years in both groups, and that a “fair” localization process between the (acoustic) normal-hearing ear and the (electric) ear with a CI is only effective with the omnidirectional setting (when testing in a semi-circle); all other MD settings would influence the area of perception too much.

This study was not without limitations. First, like most studies on CI users, more participants would have been beneficial. Second, regarding SONNET users, the questionnaires which they completed prior to testing were not sensitive to microphone setting. In a previously published “upgrade” SONNET study, users had a clear preference for specific microphone settings [13]. However, the published “upgrade” studies used a within-subject design, comparing two sound processors (old versus new). It might be plausible that a habituated audio processor as it was used in our investigation was no longer perceived as intrusive, which could also be interpreted as a good result. Third, 40/69 potential participants declined to be included in the study. While we do not know their reason(s) for doing so, it possible that selection bias may have influenced the results.

### Clinical implications

The results of the present study should be helpful in clinical routine to determine which CI users with SSD might benefit from an audio processor upgrade trial. In our two groups, speech performance in quiet, duration of deafness, and duration of CI use were all similar. We, therefore, cannot explicitly name a factor that could determine who would benefit more from an upgrade before starting a test trial with the SONNET. As we have learned, the biggest impact can be found by comparing the microphone directionality settings in measuring binaural effects. We, therefore, suggest conducting baseline testing with the “older” audio processor to determine binaural effects and localization abilities. The microphone settings of the SONNET should be programmed as follows: in P1 natural, P2 adaptive, and P3 omnidirectional. Furthermore, the recipient should be instructed to vary which microphone setting which they use over a trial period of 4 weeks. When they return to the clinic, binaural effects with each of the three different microphone modes should be measured to determine if there is a hearing improvement with the adaptive setting.

In the past, we upgraded many bilateral or bimodal CI users. In our experience, most users prefer the sound quality in the new sound processor; however, some users did not like the “new” sound at all. Based on our investigation on CI users with SSD, it seems that the sound quality might not be as important as in other indication groups. Nevertheless, it is still useful and so should be subjectively assessed. Questionnaires are a useful tool for this.

## Conclusion

Our results show that the choice of microphone setting impacts CI users with SSD’s level of speech understanding in noise. This benefit of using directional microphones is more evident when binaural effects and localization abilities are assessed; therefore, these measures should be included when evaluating the benefit that CI users with SSD derive from using an audio processor with microphone directionality (as compared to one with only omnidirectionality). Among people with SSD, this could also be used to underline the benefit of CI use, as compared to not using a CI.
