# Correction to: The impact of cochlear implant microphone settings on the binaural hearing of experienced cochlear implant users with single-sided deafness

**DOI:** 10.1007/s00405-021-06619-6

**Published:** 2021-01-28

**Authors:** Anja Kurz, Maren Zanzinger, Rudolf Hagen, Kristen Rak

**Affiliations:** Department of Oto-Rhino-Laryngology, Plastic, Aesthetic and Reconstructive Head and Neck Surgery, Comprehensive Hearing Center, University Hospital Würzburg, Josef Schneider Str. 11, 97080 Würzburg, Germany

## Correction to: European Archives of Oto-Rhino-Laryngology 10.1007/s00405-020-06450-5

In the original publication of the article, under the section Binaural effects, the following sentence “*Binaural Squelch* With the SONNET, the head shadow effect was − 1.2 dB (range − 5.9‒0.6) in natural mode, 0.9 dB (range − 4.3‒6.1), and in adaptive mode, and − 0.4 dB (range − 4.4‒2.5) in the omnidirectional mode” was published incorrectly.

The correct sentence should read as “*Binaural Squelch* With the SONNET, the binaural squelch effect was − 1.2 dB (range − 5.9‒0.6) in natural mode, 0.9 dB (range − 4.3‒6.1), and in adaptive mode, and − 0.4 dB (range − 4.4‒2.5) in the omnidirectional mode.”

In addition, there are some mistakes in the paragraph “*Binaural Summation*”. The correct paragraph should read as below,

*Binaural Summation* With the SONNET, the binaural summation effect was − 0.6 dB (range − 1.1‒2.7) in natural mode, 0.0 dB (range − 1.3‒2.2), and in adaptive mode, and 0.2 dB (range − 1.2‒2.1) in the omnidirectional mode. No significant differences were found between SONNET modes. With the OPUS 2/RONDO, the binaural summation effect was − 0.4 dB (range − 1.1‒1.6). No significant differences were found between SONNET modes and the OPUS 2/RONDO. (see Fig. 2c).

The original article has been updated.

